# Context-dependent pharmacological effects of GLP-1 receptor agonists in breast cancer from molecular mechanisms to clinical safety

**DOI:** 10.3389/fphar.2026.1978397

**Published:** 2026-10-28

**Authors:** Zhen-yu Xie, Yue Qian, Ting-ting Liu, Ping Xiang, Ying Mu, Wei Zhang

**Affiliations:** 1 Department of Clinical Laboratory, The Central Hospital of Qinghe County, Xingtai, Hebei, China; 2 Department of Pathogen Biology, Guizhou Nursing Vocational College, Guiyang, Guizhou, China; 3 Department of Clinical Laboratory, Zunyi Hospital of Traditional Chinese Medicine, Zunyi, Guizhou, China; 4 Medical Affairs Department, The Second Affiliated Hospital of Zunyi Medical University, Zunyi, Guizhou, China

**Keywords:** breast cancer, breast cancer molecular subtypes, drug safety, GLP-1 receptor agonists, metabolic regulation, tumor microenvironment

## Abstract

Glucagon-like peptide-1 receptor agonists (GLP-1 RAs) have become cornerstone therapeutics for type 2 diabetes mellitus and obesity, but their impact on breast cancer remains uncertain. Preclinical studies show context-dependent effects of GLP-1 RAs on breast-cancer cells. Direct evidence from breast-cancer models supports a pro-proliferative NADPH oxidase 4 (NOX4)/reactive oxygen species (ROS) /VEGF pathway under supra-pharmacological exposure, whereas PI3K/Akt-mediated pro-survival signaling and cAMP/PKA/CREB-driven proliferation remain inferred from non-breast systems and require direct confirmation. Under obesity- and insulin-resistant conditions, indirect antitumor effects may occur through improved insulin sensitivity, altered adipokines, and remodeling of the tumor immune microenvironment. Meta-analyses of randomized controlled trials do not show a significant increase in breast-cancer risk (RR 0.98, 95% CI 0.76-1.26), while some large observational cohorts report small risk differences that are susceptible to confounding and detection bias. Evidence for inter-agent differences is observational and limited by the absence of head-to-head randomized oncological comparisons. This narrative review synthesizes preclinical and clinical evidence across three dimensions, namely, molecular subtype, drug exposure, and host metabolic status, and distinguishes direct breast-cancer evidence from mechanistic extrapolation. We outline evidence-informed clinical considerations and research priorities rather than definitive recommendations.

## 1 Introduction

Breast cancer is the most frequently diagnosed malignancy among women worldwide, and its pathogenesis is closely linked to metabolic dysfunction, particularly obesity, hyperinsulinemia, and type 2 diabetes mellitus. Data from the 2023 Global Burden of Disease (GBD) study showed that the age-standardized disability-adjusted life-year (DALY) rate for diabetes increased by 14.9% between 2010 and 2023, with high body-mass index and elevated fasting plasma glucose as major metabolic risk factors (GBD, 2024). Obesity drives breast-cancer pathogenesis through chronic low-grade inflammation, hormonal dysregulation with enhanced aromatase activity, adipokine imbalance, and hyperinsulinemia with subsequent activation of the PI3K/Akt/mTOR and MAPK pathways (Lauby-Secretan et al., 2016; Goodwin et al., 2002). Obesity is also associated with inferior outcomes in postmenopausal women with hormone-receptor-positive breast cancer, including an increased risk of recurrence during aromatase-inhibitor therapy (Harborg et al., 2023). Meta-analyses of bariatric surgery have reported reduced breast-cancer incidence (RR 0.56), indirectly supporting the cancer-preventive potential of metabolic interventions (Lim et al., 2024).

Glucagon-like peptide-1 receptor agonists, including semaglutide, liraglutide, and the dual-target glucagon-like peptide-1 (GLP-1)/glucose-dependent insulinotropic polypeptide (GIP) agonist tirzepatide, have revolutionized the management of type 2 diabetes and obesity through glucose-dependent insulin secretion, glucagon suppression, delayed gastric emptying, and appetite reduction (Ungvari et al., 2025). As their use rapidly expands to non-diabetic populations, concerns regarding oncological safety, especially for hormone-sensitive malignancies, such as breast cancer, have intensified. Existing evidence is conflicting; preclinical studies report both pro-tumor and anti-tumor effects conditional on experimental conditions, while epidemiological investigations yield inconsistent risk estimates.

Several recent reviews have broadly discussed GLP-1 RAs and cancer (Valencia-Rincón et al., 2025; Luconi et al., 2025; Miousse, 2025; Kupnicka et al., 2024), including a 2025 synthesis in The Journal of Clinical Investigation covering clinical evidence and translational opportunities for preclinical research across multiple malignancy types, and other reviews have addressed GLP-1 RA use in breast cancer from clinical or survivor perspectives (Xande and Del Giglio, 2025; Garczorz et al., 2024; Jahan et al., 2026). However, most prior reviews present evidence in parallel without an integrated framework linking three key regulatory dimensions: breast-cancer molecular subtype and its influence on GLP-1 receptor expression and downstream signaling; drug exposure, including therapeutic versus supra-pharmacological concentrations, as a determinant of signaling-pathway bias; and host metabolic status, which governs the relative contributions of indirect and direct effects. Inter-agent differences and long-term safety data also remain incompletely integrated.

This narrative review synthesizes preclinical mechanisms and clinical evidence within a three-dimensional framework of molecular subtype, drug exposure, and metabolic status. We do not assert definitive pro-tumor or anti-tumor effects. Direct evidence obtained in breast-cancer models is distinguished from mechanistic inference derived from pancreatic, cardiovascular, neurological, or other non-breast systems. The literature was identified through targeted searches of publicly available biomedical databases and citation tracking, with studies selected for relevance to GLP-1 RA biology, breast-cancer outcomes, and metabolic regulation. No formal systematic review protocol, inclusion and exclusion screening, risk-of-bias assessment, or quantitative synthesis was applied.

## 2 Biological basis of the GLP-1 receptor and its agonists

### 2.1 Tissue distribution and signaling pathways of the GLP-1 receptor

The glucagon-like peptide-1 receptor (GLP-1R) is a class B G-protein-coupled receptor consisting of 463 amino acids, featuring a large extracellular N-terminal domain and seven-transmembrane helices that primarily couple to Gs proteins (Doyle and Egan, 2007). Initially identified as a core regulator of insulin secretion in pancreatic β-cells, GLP-1R is now known to be widely expressed in the gastrointestinal tract, heart, kidney, lung, central nervous system, and multiple peripheral tissues (Müller et al., 2019; Tornehave et al., 2008). Within the gastrointestinal tract, GLP-1R is present in gastric mucous neck cells, duodenal Brunner’s glands, enteroendocrine cells, intraepithelial lymphocytes, and nerve fibers of the myenteric plexus, indicating its roles in gastrointestinal motility, mucosal protection, and local immune modulation (Müller et al., 2019). In the kidney, GLP-1R expression in vascular smooth muscle cells and distal renal tubules is downregulated under pathological conditions such as hypertension or glomerular injury (Richards et al., 2014; Kim et al., 2013). In the cardiovascular system, GLP-1R occurs in cardiomyocytes, vascular endothelial cells, and lymphatic-endothelial cells, and its activation mediates cardioprotection and improves lymphatic-pump function (Bjørnholm et al., 2021; Nuamnaichati et al., 2022). In the liver, GLP-1R signaling contributes to the weight-loss-independent metabolic effects of semaglutide in mice (Gonzalez-Rellan et al., 2026). These findings establish a broad biological basis for GLP-1R actions but do not demonstrate direct effects in breast cancer.

GLP-1R has been detected in ER-positive MCF-7 cells (Refaat et al., 2026)and in triple-negative MDA-MB-231 and MDA-MB-468 cells (Liu et al., 2022). However, these observations come from a small number of cell lines and experimental systems. The available data do not consistently show that breast-cancer cells express higher GLP-1R levels than normal mammary epithelium, and comprehensive expression data across clinical molecular subtypes remain limited. Upon activation, GLP-1R couples primarily to Gs, increasing cAMP and activating PKA- and Epac-dependent signaling (Doyle and Egan, 2007; Liu et al., 2022). PI3K/Akt and ERK1/2 activation have been described in pancreatic and other non-breast systems and are therefore presented here as indirect mechanisms (Madsbad, 2016). In breast-cancer cells (Figure 1), direct evidence supports GLP-1R-dependent activation of the NOX4/ROS/VEGF pathway under the experimental conditions described below (Liu et al., 2022).

**FIGURE 1:**
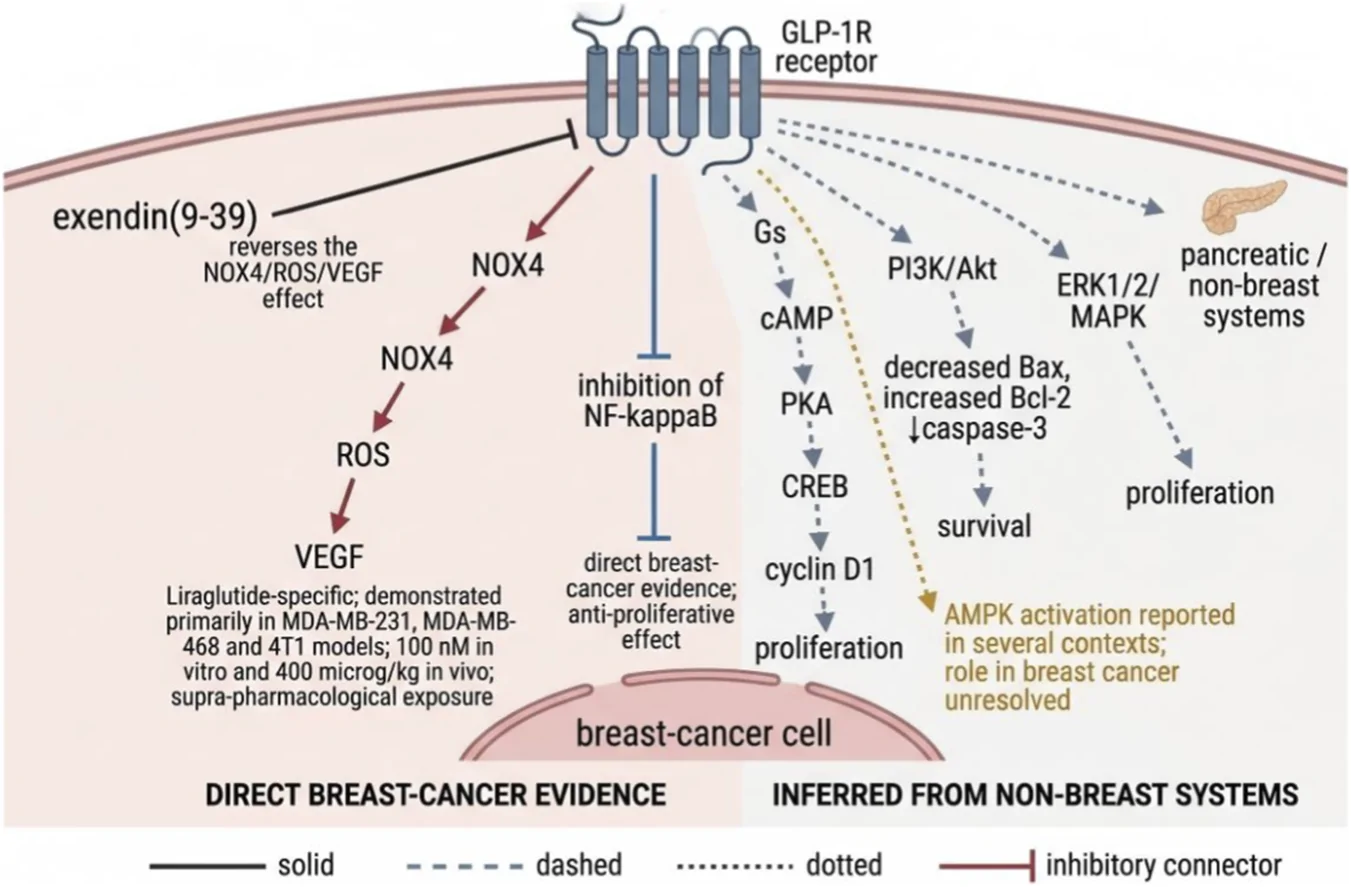
GLP-1R signaling in breast-cancer cells and evidence boundaries.

### 2.2 Pharmacological profiles and inter-agent differences

Currently approved GLP-1 RAs include exenatide, liraglutide, dulaglutide, semaglutide, and tirzepatide, which differ in structure, sequence similarity to human GLP-1, receptor-binding affinity, duration of action, and pharmacokinetics (Liu, 2024; Derington et al., 2025; Moiz et al., 2026). These pharmacological differences may influence metabolic efficacy and adverse-effect profiles, but they do not by themselves establish oncological superiority or safety, and pathway-specific generalizations should be avoided without direct experimental evidence for each agent.

Cardiovascular and metabolic outcomes differ across agents. In a comparative cohort of patients with type 2 diabetes, semaglutide and liraglutide were associated with lower risks of major adverse cardiovascular events than dulaglutide (Derington et al., 2025), and blood-pressure effects have also been reviewed (Moiz et al., 2026). Semaglutide improved histological and metabolic outcomes in nonalcoholic steatohepatitis in a randomized trial (Newsome et al., 2021). Cancer-risk comparisons between agents are observational. A meta-analysis of randomized trials found no significant increase in breast-cancer risk (RR 0.98, 95% CI 0.76-1.26) (Piccoli et al., 2021), and thyroid-cancer safety signals require continued monitoring (Hu et al., 2022). These data do not support selecting one GLP-1 RA over another on oncological grounds.

Tirzepatide, a dual GLP-1/GIP-receptor agonist, produces greater weight loss and glycemic control than mono-target GLP-1 RAs and has distinct cardiometabolic pharmacology (Fisman and Tenenbaum, 2021). In a preclinical study using an obesity-driven endometrial cancer mouse model, tirzepatide reduced body weight and suppressed tumor growth by modulating glycolysis, fatty acid metabolism, and cytokine/chemokine production in the tumor microenvironment (Kong et al., 2024). These findings are derived from preclinical models and should not be extrapolated to patients, as no breast-cancer-specific clinical outcomes are currently available. GLP-1 RAs also modulate microRNA expression in non-cancer systems (Miao et al., 2025), and GLP-1R signaling may influence immune-cell function (Frey, 2026). These effects provide plausible indirect routes to tumor-microenvironment remodeling, but direct validation in breast-cancer models is limited (Figure 2).

**FIGURE 2:**
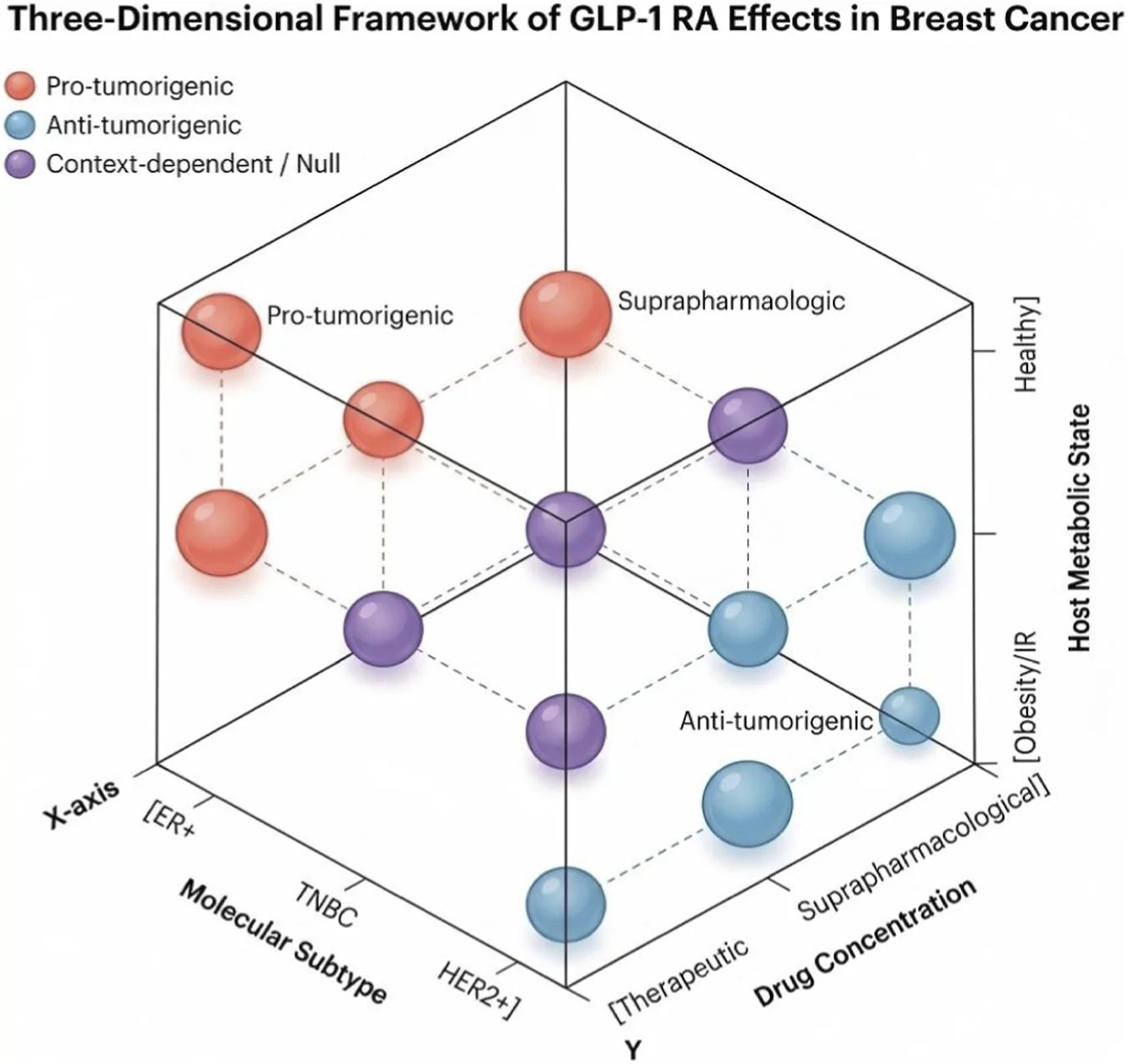
Three-dimensional framework for GLP-1-RA effects in breast cancer.

## 3 Direct effects of GLP-1 receptor agonists on breast-cancer-cell proliferation

### 3.1 Pro-proliferative mechanisms and conditional evidence

GLP-1 RAs may promote breast-cancer cell proliferation under specific experimental conditions, but most supporting evidence comes from *in vitro* studies using concentrations above typical clinical exposure. In this review, therapeutic exposure denotes concentrations or doses within approved human dosing ranges or supported by human pharmacokinetic data. Supra-pharmacological exposure denotes experimental conditions exceeding those ranges unless equivalence has been demonstrated.

Concentration-dependent effects have been reported. In a direct *in vitro* study, liraglutide stimulated MDA-MB-231 growth and increased ATP-binding cassette transporter expression, with toxicity observed only at concentrations far above serum liraglutide concentrations (Shadboorestan et al., 2021). At higher concentrations (100 nM *in vitro*; 400 μg/kg *in vivo*), liraglutide activated GLP-1R and increased NOX4, ROS, and VEGF signaling in triple-negative breast-cancer models; the effect was reversed by the GLP-1R antagonist exendin (Miousse, 2025; Kupnicka et al., 2024; Xande and Del Giglio, 2025; Garczorz et al., 2024; Jahan et al., 2026; Doyle and Egan, 2007; Müller et al., 2019; Tornehave et al., 2008; Richards et al., 2014; Kim et al., 2013; Bjørnholm et al., 2021; Nuamnaichati et al., 2022; Gonzalez-Rellan et al., 2026; Refaat et al., 2026; Liu et al., 2022; Madsbad, 2016; Liu, 2024; Derington et al., 2025; Moiz et al., 2026; Newsome et al., 2021; Piccoli et al., 2021; Hu et al., 2022; Fisman and Tenenbaum, 2021; Kong et al., 2024; Miao et al., 2025; Frey, 2026; Shadboorestan et al., 2021; Drucker, 2018; Kang et al., 2006; Iwaya et al., 2017; Tanaka et al., 2021) (23). The 100 nM *in vitro* concentration exceeds typical clinical exposure and is therefore classified as supra-pharmacological. cAMP/PKA/CREB-driven cyclin-D1 upregulation has been described in pancreatic beta cells and other non-breast systems (Doyle and Egan, 2007; Drucker, 2018; Kang et al., 2006), but direct evidence in breast-cancer cells is lacking. Similarly, PI3K/Akt-mediated Bax inhibition, Bcl-2 upregulation, and caspase-3 suppression are inferred from general GLP-1R biology rather than demonstrated in breast-cancer models.

Evidence is also skewed across molecular subtypes. Pro-proliferative findings originate mainly from triple-negative (MDA-MB-231, 4T1) and ER-positive (MCF-7) models (Liu et al., 2022; Shadboorestan et al., 2021; Iwaya et al., 2017), whereas HER2-positive and other subtypes are under-represented. Differences in GLP-1R expression and downstream signaling may therefore determine response direction.

### 3.2 Anti-proliferative mechanisms and conditional evidence

Anti-proliferative effects have been reported in specific breast-cancer models. Exendin-4 attenuated MCF-7 and MDA-MB-231 growth by inhibiting NF-kappaB activation (Iwaya et al., 2017), and exendin-4 plus metformin additively reduced proliferation, tumor weight, and Ki-67-positive cells in xenograft models (Tanaka et al., 2021). Claims that receptor desensitization or AMPK activation universally mediates these effects remain indirect and require breast-cancer-specific validation (Shadboorestan et al., 2021; Zhang et al., 2025).

In MCF-7 cells cultured in obese adipose tissue-derived conditioned medium, liraglutide inhibited proliferation, reduced colony formation, and induced G0/G1 arrest while modulating glycolysis, oxidative stress, and adipokine expression (Alanteet et al., 2021). These findings support context-dependent anti-proliferative effects in obesity-associated breast cancer, but they derive from a limited number of *in vitro* models and should not be generalized to all breast-cancer subtypes. Liraglutide decreased leptin and inflammatory mediator expression and increased adiponectin expression in adipocytes and breast-cancer cells, providing a plausible mechanism for indirect anti-proliferative effects (Alanteet et al., 2021). This pathway has not been confirmed in prospective clinical studies.

## 4 Effects of GLP-1 receptor agonists on breast-cancer metastasis and invasion

### 4.1 Pro-metastatic mechanisms and limitations of evidence

Direct experimental evidence for GLP-1 RA-induced breast-cancer metastasis remains limited. Several mechanistic assertions rely on indirect inference or studies performed in other tumor types.

Changes in MMP-2, MMP-9, migration, and invasion have been reported in some aggressive breast-cancer models, but the findings have not been consistently replicated. GLP-1 signaling may have context-dependent oncogenic or anti-oncogenic effects, and net outcomes are likely determined by receptor expression, tumor microenvironment, and downstream signaling (Iwaya et al., 2017). NF-kappaB- and STAT3-dependent epithelial-to-mesenchymal transition has been proposed from non-breast cellular systems. In breast-cancer cells, the best direct evidence concerns NF-kappaB inhibition by exendin-4 (Iwaya et al., 2017). Reports of increased pulmonary metastatic burden after long-term GLP-1 RA exposure require replication and careful dose-response characterization before they can be considered established.

Overall, MMP upregulation, NF-kappaB/STAT3-mediated epithelial-to-mesenchymal transition, and *in vivo* promotion of lung metastasis currently have limited high-quality direct support in breast cancer (Iwaya et al., 2017). These mechanisms should be presented as hypotheses and research gaps.

### 4.2 Anti-metastatic mechanisms

Compared with the limited pro-metastatic evidence, anti-metastatic mechanisms receive stronger experimental and theoretical support, operating predominantly through indirect metabolic and immunomodulatory pathways.

GLP-1 RAs improve insulin sensitivity and reduce hyperinsulinemia, which may indirectly lower IGF-1 signaling. IGF-1R activation promotes breast-cancer resistance and metastatic signaling through PI3K/Akt and MAPK/ERK (Ianza et al., 2021). The proposed effect of GLP-1 RAs on this axis in breast cancer remains indirect and requires direct measurement of circulating and intratumoral IGF-1 pathway activity.

The breast tumor microenvironment contains adipocytes, fibroblasts, and immune cells that can influence tumor progression. GLP-1R expression has been most clearly documented in breast-cancer cells. In adipocytes, GLP-1 RA treatment can alter leptin, adiponectin, and inflammatory mediator secretion, providing a plausible route for indirect effects on tumor cells (Alanteet et al., 2021). GLP-1 RAs can also modulate macrophage phenotype in inflammatory or immunotherapy contexts, but direct evidence in breast tumors is limited (Shadboorestan et al., 2021). We found no consistent primary evidence that breast cancer-associated fibroblasts express functional GLP-1R. Effects on stromal cells are therefore plausible but remain largely indirect and require cell-type-specific validation. Leptin activates JAK2/STAT3 and PI3K/Akt signaling and promotes breast-cancer progression, and GLP-1 RA-mediated reductions in adipose leptin may contribute to indirect antitumor effects, although direct metastasis-specific evidence remains limited (Alanteet et al., 2021; Sánchez-Jiménez et al., 2019).

## 5 Metabolic regulation and the breast-tumor microenvironment

### 5.1 Improvement of insulin resistance and hyperinsulinemia

Diabetes and obesity are associated with increased cancer risk (Szablewski, 2014). Hyperinsulinemia is a potential driver of breast-cancer progression, particularly in patients with obesity or type 2 diabetes. Chronic hyperinsulinemia can activate insulin receptor and IGF-1R signaling, promote proliferation, survival, and migration (Goodwin et al., 2002; Valencia-Rincón et al., 2025), and amplify PI3K/Akt and MAPK/ERK signaling (Hu et al., 2020). These mechanisms provide a rationale for indirect antitumor effects of GLP-1 RAs but do not establish causality in breast cancer.

GLP-1 RAs improve systemic insulin sensitivity through glucose-dependent insulin secretion, glucagon suppression, weight loss, and reduced visceral adiposity (Liu, 2024; Vilsbøll et al., 2012). In metabolic disease populations, semaglutide and other GLP-1 RAs reduce HOMA-IR and improve insulin sensitivity (Niu et al., 2025). The magnitude and oncological relevance of these changes in breast-cancer patients remain uncertain.

GLP-1 RA treatment reduces fasting insulin and HOMA-IR in metabolic disease populations (Liu, 2024; Vilsbøll et al., 2012), but the relationship between these changes and breast-cancer biomarkers such as Ki-67 has not been prospectively established.

### 5.2 Weight-loss-mediated remodeling of the tumor microenvironment

Obesity promotes a pro-tumorigenic microenvironment through adipokine imbalance, chronic low-grade inflammation, and increased aromatase activity, which raises estrogen bioavailability (Lauby-Secretan et al., 2016; Sánchez-Jiménez et al., 2019). These pathways are particularly relevant to hormone-receptor-positive breast cancer.

GLP-1 RAs induce weight loss and reduce visceral fat (Vilsbøll et al., 2012; Moore et al., 2023), which may lower adipose-derived leptin and estrogen and improve insulin sensitivity (Lauby-Secretan et al., 2016; Sánchez-Jiménez et al., 2019). However, direct evidence that these systemic changes alter the human breast-tumor immune microenvironment is limited. Proposed effects on macrophage polarization and CD8^+^ T Cell function are largely extrapolated from non-breast disease models (Frey, 2026; Hundal et al., 2026). Direct evidence from breast-cancer biopsies during GLP-1 RA treatment remains sparse (Figure 3). Prospective studies with paired tumor tissue, immune profiling, and metabolic endpoints are needed before systemic metabolic changes can be interpreted as breast-cancer-specific immunomodulation (Hundal et al., 2026).

**FIGURE 3:**
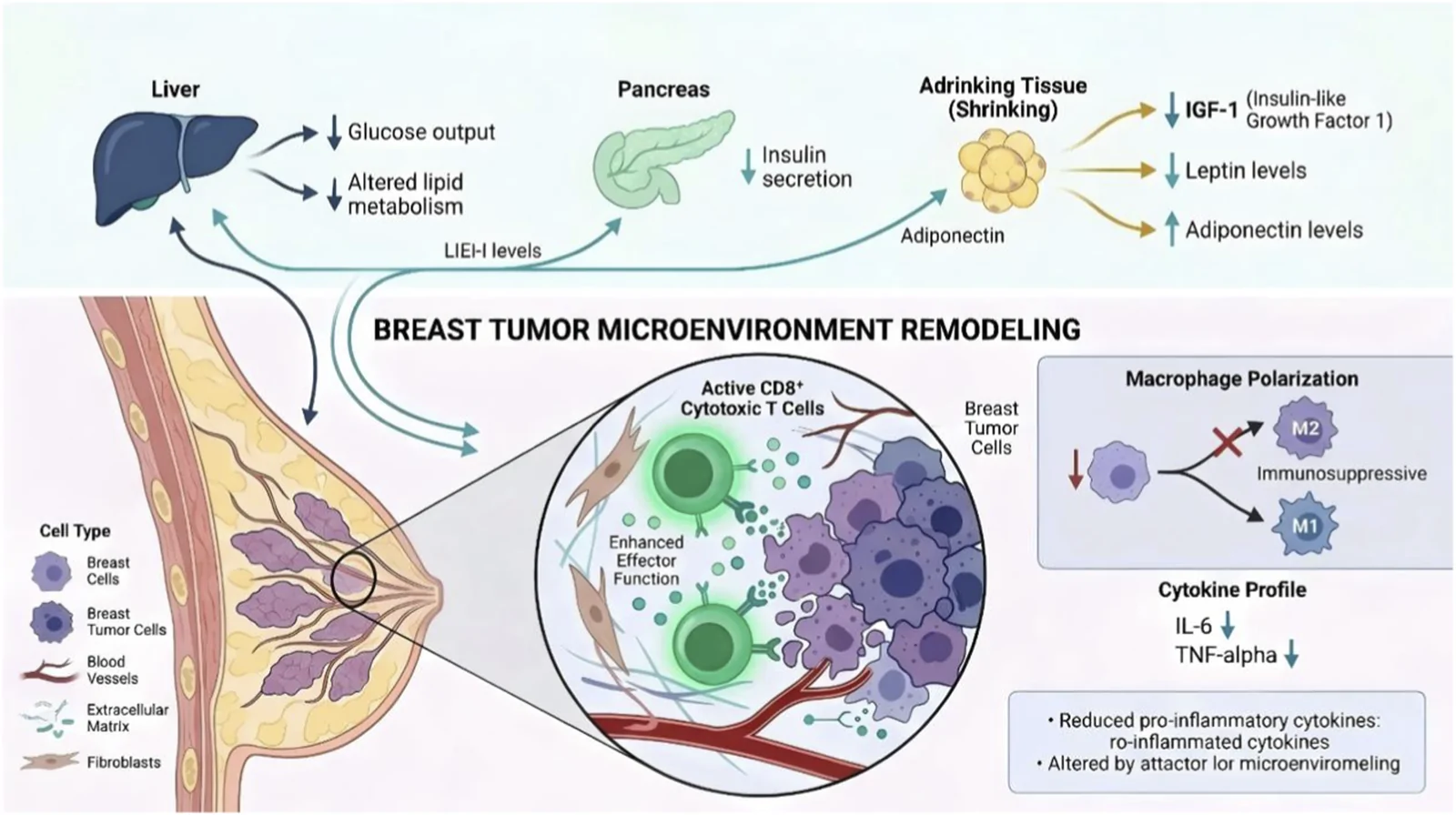
Multi-scale paradigm of systemic metabolic regulation and breast tumor immune microenvironment remodeling mediated by GLP-1 RAs.

## 6 Pre-clinical evidence and animal models

### 6.1 Xenograft models

Effects of GLP-1 RAs in breast-cancer models are heterogeneous and depend on molecular subtype, drug exposure, and model system (Table 1). Direct evidence is available mainly from cell lines and xenografts, whereas findings from pancreatic, cardiovascular, and neurological systems should be treated as indirect. Pro-tumorigenic findings: In a triple-negative breast-cancer model, high-dose liraglutide increased tumor progression through GLP-1R-dependent NOX4/ROS/VEGF signaling, and the effect was reversed by exendin (Miousse, 2025; Kupnicka et al., 2024; Xande and Del Giglio, 2025; Garczorz et al., 2024; Jahan et al., 2026; Doyle and Egan, 2007; Müller et al., 2019; Tornehave et al., 2008; Richards et al., 2014; Kim et al., 2013; Bjørnholm et al., 2021; Nuamnaichati et al., 2022; Gonzalez-Rellan et al., 2026; Refaat et al., 2026; Liu et al., 2022; Madsbad, 2016; Liu, 2024; Derington et al., 2025; Moiz et al., 2026; Newsome et al., 2021; Piccoli et al., 2021; Hu et al., 2022; Fisman and Tenenbaum, 2021; Kong et al., 2024; Miao et al., 2025; Frey, 2026; Shadboorestan et al., 2021; Drucker, 2018; Kang et al., 2006; Iwaya et al., 2017; Tanaka et al., 2021) (23). Liraglutide also promoted MDA-MB-231 growth and increased ABC-transporter expression *in vitro* (Shadboorestan et al., 2021). These effects occurred at or above supra-pharmacological exposure and require cautious interpretation.

**TABLE 1:** Summary of pre-clinical evidence for GLP-1 RAs in breast-cancer models.

Study (Year)	Agent	Exposure	Model/duration	Cell line(s)/subtype	Effect direction	Key mechanism	Evidence boundary
(Liu et al., 2022)	Liraglutide	100 nM *in vitro*; 400 μg/kg *in vivo*	*In vitro*; xenograft/14 days	MDA-MB-231, MDA-MB-468; 4T1	Pro-tumorigenic	GLP-1R-dependent NOX4/ROS/VEGF signaling	Direct; reversed by exendin (Miousse, 2025; Kupnicka et al., 2024; Xande and Del Giglio, 2025; Garczorz et al., 2024; Jahan et al., 2026; Doyle and Egan, 2007; Müller et al., 2019; Tornehave et al., 2008; Richards et al., 2014; Kim et al., 2013; Bjørnholm et al., 2021; Nuamnaichati et al., 2022; Gonzalez-Rellan et al., 2026; Refaat et al., 2026; Liu et al., 2022; Madsbad, 2016; Liu, 2024; Derington et al., 2025; Moiz et al., 2026; Newsome et al., 2021; Piccoli et al., 2021; Hu et al., 2022; Fisman and Tenenbaum, 2021; Kong et al., 2024; Miao et al., 2025; Frey, 2026; Shadboorestan et al., 2021; Drucker, 2018; Kang et al., 2006; Iwaya et al., 2017; Tanaka et al., 2021); supra-pharmacological exposure
(Shadboorestan et al., 2021)	Liraglutide	Concentrations not mapped to human exposure	*In vitro*/not stated	MDA-MB-231 (TNBC)	Pro-proliferative and pro-migratory	AMPK-dependent EMT; ABC transporter upregulation	Direct *in vitro* evidence only
(Alanteet et al., 2021)	Liraglutide	Concentrations not reported in abstract	*In vitro* obese adipose tissue-conditioned medium	MCF-7 (ER+)	Anti-proliferative	Leptin downregulation; adiponectin upregulation; G0/G1 arrest	Direct *in vitro* evidence in an obesity-associated model
(Refaat et al., 2026)	Liraglutide	Concentrations not reported in abstract	*In vitro*	MCF-7 (ER+)	Anti-proliferative	Glycolysis, oxidative stress, and adipokine modulation	Direct *in vitro* evidence; prostate-cancer arm is outside this review
(Iwaya et al., 2017)	Exendin-4	Not reported in abstract	*In vitro* and xenograft	MCF-7, MDA-MB-231	Anti-proliferative and anti-tumorigenic	NF-kappaB inhibition	Direct breast-cancer evidence
(Tanaka et al., 2021)	Exendin-4 plus metformin	10 nM exendin-4; 0.1 mM metformin	*In vitro* and xenograft	MCF-7, MDA-MB-231, KPL-1	Additive anti-tumor effect	Reduced proliferation; metformin-associated apoptosis via BCL-2	Direct breast-cancer evidence; not semaglutide-specific

Abbreviations: ABC, ATP-binding cassette; ADSC, adipose-derived stem cell; AMPK, AMP-activated protein kinase; EMT, epithelial-to-mesenchymal transition; ER, estrogen receptor; GLP-1R, glucagon-like peptide-1, receptor; NF-kappaB, nuclear factor kappa B; NOX4, NADPH, oxidase 4; ROS, reactive oxygen species; TNBC, triple-negative breast cancer; VEGF, vascular endothelial growth factor. Supra-pharmacological exposure denotes concentrations or doses exceeding approved human exposure ranges unless equivalence has been demonstrated.

Anti-tumorigenic findings: Exendin-4 attenuated MCF-7 and MDA-MB-231 growth by inhibiting NF-kappaB activation (Iwaya et al., 2017). In a separate xenograft study, exendin-4 plus metformin reduced tumor weight and Ki-67-positive cells, with metformin contributing primarily to apoptosis (Tanaka et al., 2021). We did not identify sufficiently robust direct evidence for a semaglutide-specific reduction in breast-cancer metastasis, and we have therefore removed that claim.

Together, these findings indicate that GLP-1 RA effects depend on molecular subtype, GLP-1R expression, exposure level, and the tumor microenvironment. They do not support a class-wide pro-tumorigenic or anti-tumorigenic conclusion.

### 6.2 Genetically-modified models and research gaps

Direct studies using GLP-1R-knockout breast-cancer models remain extremely limited. Based on GLP-1R signaling in other tissues, GLP-1R deficiency could alter proliferation or immune surveillance, but this remains an untested hypothesis in breast cancer (Ungvari et al., 2025; Drucker, 2018).

Interventional studies with GLP-1 RAs in MMTV-PyMT or other spontaneous breast-cancer models are scarce (Xande and Del Giglio, 2025). Whether GLP-1 RAs primarily influence early tumorigenesis through improved insulin sensitivity and reduced inflammation remains speculative. Available animal evidence is largely confined to cell-line xenografts and a small number of diet-induced obesity models (Liu et al., 2022; Shadboorestan et al., 2021; Iwaya et al., 2017; Tanaka et al., 2021). The scarcity of immunocompetent spontaneous breast-cancer models is a key limitation for mechanistic and translational inference.

## 7 Clinical trials and real-world evidence

This section summarizes clinical evidence by study design, from randomized trials and meta-analyses to large observational cohorts and Mendelian randomization. Evidence strength is interpreted according to design, confounding control, follow-up, and whether breast-cancer outcomes were prespecified (Table 2).

**TABLE 2:** Summary of clinical evidence for GLP-1 RAs and breast-cancer risk/outcomes.

Study (Year)	Design	Population	Exposure/comparator	Breast-cancer finding	Main limitation
(Hicks et al., 2016)	Population-based cohort (CPRD)	44,984 women with T2DM	GLP-1 analogues vs. DPP-4 inhibitors	Overall HR 1.40 (0.91-2.16); transient signal at years 2–3	Observational; detection bias may explain the transient signal
(Santella et al., 2020)	Population-based cohort	Obese women with diabetes	GLP-1 RA-associated weight loss	Weight loss may increase breast-cancer detection	Supports detection bias rather than causality
(Wang et al., 2024)	Retrospective EHR cohort	1,651,452 patients with T2DM	GLP-1 RAs vs. insulin or metformin	No increase in postmenopausal breast cancer; lower risk for several other obesity-related cancers	Observational; residual confounding
(Dai et al., 2025)	Retrospective cohort	1.1 million adults with obesity	GLP-1 RAs	Lower breast-cancer risk estimate; semaglutide and liraglutide subgroups differed	No head-to-head randomized comparison; confounding by indication
(Gamborg et al., 2025)	Nationwide emulated trial	Danish nationwide initiators	GLP-1 RAs vs. DPP-4 inhibitors	Overall cancer HR 1.35 (1.05-1.73) during years 6–10	Survival bias and residual BMI confounding
(Piccoli et al., 2021)	Meta-analysis of RCTs	52 trials with breast-cancer events	GLP-1 RAs vs. control	RR 0.98 (0.76-1.26)	Trials not powered for cancer; short follow-up
(Ko et al., 2026)	Meta-analysis of RCTs	48 trials; 94,245 participants	GLP-1 RAs or dual agonists vs. control	OR 0.95 (0.60-1.49)	Low event counts and limited follow-up
(Wolff Sagy et al., 2025)	Retrospective cohort (Clalit)	Adults with obesity and T2DM	First-generation GLP-1 RAs vs. bariatric surgery	Obesity-related cancer HR 1.11 (0.86-1.44)	Observational; breast cancer not separately reported
(Den et al., 2026)	Retrospective cohort (TriNetX)	Postmenopausal women with obesity and breast cancer	GLP-1 RAs vs. bariatric surgery	Overall survival HR 0.57; recurrence HR 0.52	Selection bias; observational design
(Sun et al., 2024)	Drug-target MR and clinical trials	Genetically proxied GLP-1R exposure	GLP-1R agonism	No consistent increase in overall cancer risk; limited breast-cancer-specific estimates	Lifelong genetic exposure differs from pharmacological treatment

Abbreviations: BMI, body-mass index; CI, confidence interval; CPRD, clinical practice research datalink; DPP-4, dipeptidyl peptidase-4; EHR, electronic health record; GLP-1, RA, glucagon-like peptide-1, receptor agonist; HR, hazard ratio; MR, mendelian randomization; OR, odds ratio; RCT, randomized controlled trial; RR, risk ratio; T2DM, type 2 diabetes mellitus.

### 7.1 Safety end-points from randomized controlled trials

Prespecified cancer endpoints from randomized trials provide the strongest available causal inference, although follow-up is short and event counts are low. A meta-analysis of 52 randomized trials found no significant increase in breast-cancer risk (RR 0.98, 95% CI 0.76-1.26) (Piccoli et al., 2021). A more recent systematic review and meta-analysis of 48 trials similarly found little or no effect on breast-cancer risk (OR 0.95, 95% CI 0.60-1.49) (Ko et al., 2026). These estimates are imprecise because the trials were not designed to evaluate cancer outcomes.

Existing randomized trials were not powered for breast-cancer endpoints, mean follow-up was generally 2–5 years, and breast-cancer events were uncommon. Longer-term and adequately powered studies are therefore needed.

### 7.2 Large-scale cohorts and real-world investigations

Hicks et al. used the UK Clinical Practice Research Datalink to compare GLP-1 analogues with DPP-4 inhibitors among 44,984 women with type 2 diabetes. Overall breast-cancer risk was not significantly increased (HR 1.40, 95% CI 0.91-2.16), although a transient signal was observed during years 2–3 of exposure (Hicks et al., 2016). Subsequent work suggested that this temporal pattern may reflect increased detection after weight loss rather than a causal increase in tumor incidence (Santella et al., 2020).

Wang et al. analyzed electronic health-record data from 1,651,452 patients with type 2 diabetes and reported no increase in postmenopausal breast-cancer risk with GLP-1 RA exposure, alongside reduced risk estimates for several other obesity-associated cancers (Wang et al., 2024). Dai et al. studied 1.1 million adults with obesity and reported a lower breast-cancer risk estimate with GLP-1 RA use; subgroup analyses suggested differences between semaglutide and liraglutide (Dai et al., 2025). These observational findings may be affected by confounding by indication, detection bias, and treatment duration. A retrospective Israeli cohort compared first-generation GLP-1 RAs with bariatric metabolic surgery over a median follow-up of 7.5 years. No statistically significant difference in obesity-related cancers was observed (adjusted HR 1.11, 95% CI 0.86-1.44), and weight loss explained part of the risk reduction (Wolff Sagy et al., 2025).

A TriNetX-based study compared GLP-1 RAs with bariatric surgery among postmenopausal women with obesity and breast cancer. The GLP-1 RA group had better overall survival (HR 0.57) and lower locoregional recurrence (HR 0.52), but the observational design and potential selection bias preclude causal interpretation (Den et al., 2026). Gamborg et al. conducted a Danish nationwide emulated trial and found a modest increase in overall cancer risk during years 6–10 of sustained GLP-1 RA use (HR 1.35, 95% CI 1.05-1.73). The authors noted that this signal may be influenced by survival bias and residual confounding by body-mass index (Gamborg et al., 2025).

### 7.3 Pharmacovigilance and mendelian randomization

We did not identify a breast-cancer-specific FAERS analysis that met our inclusion criteria, and we have therefore removed the previous reporting-odds-ratio estimate. Pharmacovigilance data are more appropriately used for signal detection than causal inference.

Two drug-target Mendelian randomization studies did not provide consistent evidence of increased overall cancer risk and reported limited breast-cancer-specific estimates (Sun et al., 2024; Zhou et al., 2026). A separate meta-analysis of randomized trials of semaglutide also found no clear increase in overall neoplasm risk (Zhong et al., 2026). Mendelian randomization reduces some forms of confounding and reverse causation, but lifelong genetically proxied exposure is not equivalent to short-term pharmacological treatment, and instrumental-variable assumptions must be satisfied.

### 7.4 Evidence synthesis and resolution of apparent contradictions

Apparent contradictions in clinical evidence may reflect differences in follow-up duration, comparator selection, population risk, individual drug exposure, and confounding. Long-term observational signals can be influenced by detection and survival bias (Santella et al., 2020; Dai et al., 2025; Gamborg et al., 2025), whereas randomized trials are limited by short follow-up and low event counts (Piccoli et al., 2021; Ko et al., 2026). Risk estimates also vary according to whether the comparator is DPP-4 inhibitors, insulin, or bariatric surgery (Wang et al., 2024; Wolff Sagy et al., 2025; Den et al., 2026). These factors should be considered when interpreting breast-cancer risk. A further unresolved issue is whether observed antitumor effects are caused by direct GLP-1R signaling in tumor or microenvironmental cells or by indirect systemic changes in weight, insulin sensitivity, adipokines, and inflammation. Current studies rarely use pair-fed or weight-matched controls, host-versus-tumor GLP-1R deletion, or cell-type-specific knockout models that would separate these mechanisms. Such designs are needed before direct and indirect effects can be quantified.

## 8 Potential value and safety considerations for GLP-1 receptor agonists in breast-cancer management

### 8.1 Metabolic management and survivor-associated benefits

For breast-cancer patients with obesity or type 2 diabetes, GLP-1 RAs may improve glycemic control, weight, and cardiovascular risk factors. Breast-cancer-specific observational data suggest clinically meaningful but attenuated weight loss and have not identified a unique breast-cancer toxicity to date (Hundal et al., 2026). These potential benefits require prospective confirmation, and treatment decisions should be based on metabolic indications and individualized oncological care rather than presumed antitumor effects.

### 8.2 Potentially at-risk populations and monitoring recommendations

Current evidence does not support GLP-1 RA-specific intensification of breast-cancer surveillance or preferential selection of semaglutide for oncological safety. Screening should follow established breast-cancer guidelines. Individuals with a family history, BRCA pathogenic variants, prior atypia, or carcinoma *in situ* should receive risk-appropriate care, but GLP-1 RA use should not by itself change screening intervals or the choice of agent (Hundal et al., 2026; Ko et al., 2026; Lo et al., 2026).

### 8.3 Evidence gap for male breast cancer

All the evidence synthesized in this review was derived from female populations. Although rare (approximately 1% of all cases), male breast cancer is predominantly ER-positive, associated with BRCA2 mutations, and may possess distinct metabolic and biological characteristics. Safety data for GLP-1 RAs in male breast cancer are entirely absent, constituting a clear research gap.

## 9 Discussion

This narrative review shows that GLP-1 RA effects in breast cancer are context-dependent and that clinical evidence does not support a class-wide increase in breast-cancer risk. Direct breast-cancer evidence supports a concentration-dependent NOX4/ROS/VEGF pathway under supra-pharmacological exposure (Liu et al., 2022), whereas PI3K/Akt-mediated survival signaling remains extrapolated from non-breast systems. Randomized trials and meta-analyses provide no clear increase in breast-cancer risk (Piccoli et al., 2021; Ko et al., 2026), but long-term observational signals and inter-agent differences require cautious interpretation (Dai et al., 2025; Gamborg et al., 2025).

First, GLP-1 RAs do not exert unidirectional effects on breast cancer. Under supra-pharmacological exposure or in selected triple-negative models, direct evidence supports pro-tumorigenic NOX4/ROS/VEGF signaling (Liu et al., 2022). In contrast, exendin-4 and exendin-4 plus metformin reduced tumor growth in other breast-cancer models (Iwaya et al., 2017; Tanaka et al., 2021). Indirect metabolic and immunomodulatory effects may dominate under obesity and insulin resistance, but direct evidence in breast cancer remains limited (Frey, 2026; Hundal et al., 2026). Second, current clinical evidence does not indicate a substantial class-wide increase in breast-cancer risk, although long-term observational signals and differences between agents require cautious interpretation. Third, semaglutide, liraglutide, exendin-4, and tirzepatide differ in pharmacology and metabolic potency and should not be treated as interchangeable for oncological safety.

This study has several limitations. It is a narrative review, not a systematic review. A formal search protocol, inclusion and exclusion screening, risk-of-bias assessment, and quantitative synthesis were not applied. Most direct breast-cancer evidence is preclinical, exposure conditions are heterogeneous, and clinical data are largely observational. These limitations constrain causal inference.

For clinical practice, GLP-1 RAs should be prescribed for approved metabolic indications, with breast-cancer risk managed through guideline-based screening rather than GLP-1 RA-specific surveillance. There is insufficient evidence to recommend a specific GLP-1 RA on oncological grounds (Hundal et al., 2026; Ko et al., 2026; Dai et al., 2025). Patients with established breast cancer should receive individualized multidisciplinary care, and potential interactions with cancer therapy, nutrition, and body composition should be monitored.

Future research should prioritize prospective breast-cancer-specific trials with recurrence and survival endpoints; clearly defined therapeutic versus supra-pharmacological exposure; immunocompetent spontaneous breast-cancer models; standardized assessment of GLP-1R expression across molecular subtypes; head-to-head comparisons of GLP-1 RAs and dual agonists; and direct studies of macrophage polarization, CD8^+^ T Cell function, and dose-response relationships.
